# Ecosystem services provided by spiders

**DOI:** 10.1111/brv.70044

**Published:** 2025-06-03

**Authors:** Pedro Cardoso, Stano Pekár, Klaus Birkhofer, Angela Chuang, Caroline Sayuri Fukushima, Eileen A. Hebets, Yann Henaut, Thomas Hesselberg, Jagoba Malumbres‐Olarte, Ondřej Michálek, Radek Michalko, Catherine Scott, Jonas Wolff, Stefano Mammola

**Affiliations:** ^1^ CE3C ‐ Centre for Ecology, Evolution and Environmental Changes, LIBRe ‐ Laboratory for Integrative Biodiversity Research, CHANGE ‐ Institute for Global Change and Sustainability, Faculty of Sciences University of Lisbon Lisbon 1749‐016 Portugal; ^2^ Finnish Museum of Natural History (Luomus) Pohjoinen Rautatiekatu 13, University of Helsinki Helsinki 00014 Finland; ^3^ Department of Botany and Zoology, Faculty of Science Masaryk University Kotlářská 2 Brno 611 37 Czech Republic; ^4^ Brandenburg University of Technology Cottbus‐Senftenberg Cottbus 03046 Germany; ^5^ Department of Entomology and Nematology University of Florida Lake Alfred FL 33850 USA; ^6^ Department of Environmental Science and Studies, Department of Biology Washington College Chestertown MD 21620 USA; ^7^ Biodiversity Unit University of Turku Turku 20014 Finland; ^8^ School of Biological Sciences, University of Nebraska‐Lincoln Lincoln NE 68588 USA; ^9^ Laboratorio de Conducta Animal, GAIA‐BIO, El Colegio de la Frontera Sur (ECOSUR) Av. del Centenario Km. 5.5, C.P Chetumal Quintana Roo 77014 Mexico; ^10^ Department of Biology University of Oxford South Parks Road Oxford OX1 3RB UK; ^11^ Institute for Molecular Bioscience, The University of Queensland St. Lucia QLD 4072 Australia; ^12^ Department of Forest Ecology Mendel University in Brno Zemedelska 3 Brno 613 00 Czech Republic; ^13^ Department of Natural Resource Sciences McGill University 21111 Lakeshore Road, Sainte‐Anne‐de‐Bellevue Montreal QC H9X 3V9 Canada; ^14^ Evolutionary Biomechanics, Zoological Institute and Museum, University of Greifswald Loitzer Str. 26 Greifswald 17489 Germany; ^15^ School of Natural Sciences, Macquarie University Wallumattagal Campus, Macquarie Park Sydney NSW 2109 Australia; ^16^ Molecular Ecology Group (MEG), Water Research Institute, National Research Council (CNR‐IRSA) Largo Tonolli 50 Verbania Pallanza 28922 Italy; ^17^ NBFC, National Biodiversity Future Center Palermo 90133 Italy

**Keywords:** arachnophobia, biodiversity monitoring, biomimetic technology, human well‐being, insecticides, nature‐based solutions, nutrient cycling, pathogen dispersal, pest suppression, silk

## Abstract

Spiders, ubiquitous and abundant predators in terrestrial ecosystems, often are the subjects of an unjust negative perception. However, these remarkable creatures stand as unsung heroes within our ecosystems, contributing a multitude of ecosystem services critical to human well‐being. Here, we describe the diverse spectrum of ecosystem services offered by spiders and their potential to inspire or directly provide nature‐based solutions. Provisioning services include the versatile uses of silk‐like and other materials, inspiration for biomimetic technology, medicines derived from venom, hemolymph and silk, bio‐insecticides that offer eco‐friendly alternatives to synthetic chemicals, food sources for various human communities worldwide, and unconventional yet increasingly valued pets. Regulating services provided by spiders extend to vital roles in pest suppression across diverse agricultural settings, mitigating diseases by curbing insect‐mediated pathogen dispersal, and controlling invasive species. Supporting services offered by spiders are equally extensive, involving nutrient cycling through the breakdown of organic matter, acting as food sources for predators, or creating habitats for other organisms. Beyond their tangible contributions, spiders hold a significant cultural and spiritual heritage globally and are integral to many traditional medicine practices. They inspire contemporary culture, provide educational value, contribute to mental health improvement, evoke a sense of place, offer models for scientific discovery, and are commonly employed for monitoring biodiversity and ecosystem health. To pave the way for future research, we present suggestions for exploring and quantifying the economic value of ecosystem services by spiders. While many of these services are well established and studied from various perspectives, others harbour untapped potential. Leveraging what nature inherently provides, these nature‐based solutions offer avenues to address challenges such as biodiversity erosion and societal needs. By restoring, preserving, or mimicking natural processes of spiders, we can enhance or provide essential ecosystem services, harnessing the full potential of spiders and the web of benefits they bring us.

## I. INTRODUCTION

With more than 53,000 described species worldwide (World Spider Catalog, 2025) and estimated to exceed 120,000 species (Agnarsson, Coddington & Kuntner, 2013), spiders are among the most diverse animals in terrestrial habitats. These arthropods exhibit a wide range of sizes, behaviours, and adaptations (Foelix, 2011; Mammola *et al*., 2017). From the tiny <0.5 mm body size of some Symphytognathidae species, to the massive 100 mm + Goliath birdeater (*Theraphosa blondi* (Latreille, 1804)) and the giant huntsman spider (*Heteropoda maxima* Jäger, 2001) with up to 300 mm leg span, their morphological diversity is astonishing. Some species are nomadic and cosmopolitan, while others are sedentary short‐range endemics, sometimes found only on a single island, mountain, valley, or cave, with less than 1 km^2^ range. Many spiders exhibit a unique form of long‐distance aerial dispersal *via* ballooning, using both air currents and electric fields (Morley & Robert, 2018; Montes & Gleiser, 2025). Spiders are renowned for their ability to spin silk, and the structural diversity of webs that many of them produce is remarkable. However, many species do not rely on a web for prey capture, with active hunters using different strategies making up a large proportion of species and individuals across the world (Cardoso *et al*., 2011; Wolff, Nentwig & Gorb, 2013).

Their wide range of adaptations enables spiders to thrive in virtually every terrestrial habitat on Earth. However, despite their ubiquity, many species are facing population declines and are at risk of extinction due to multiple factors, from habitat loss to invasive species, pollutants, climate change, and overexploitation (Seppälä *et al*., 2018; Branco & Cardoso, 2020). With an unknown number of species at risk, and as a group that is often either ignored or feared (Polák *et al*., 2020; Landová *et al*., 2021; Mammola *et al*., 2022), spiders are largely overlooked in conservation policy and practice (Knight, 2008; Cardoso, 2012; Mammola *et al*., 2020; Milano *et al*., 2021).

Despite the negative perception frequently held of these creatures, often associated with arachnophobia (Correia & Mammola, 2024), spiders are the unsung heroes of our ecosystems, contributing to the well‐being of the planet and humanity by providing a range of ecosystem services, i.e. benefits that humans derive from ecosystems, that often go unnoticed (e.g. Daily & Matson, 2008). These services are categorised into four main types: (I) provisioning (such as medicine and materials); (II) regulating (like biological control); (III) supporting (such as nutrient cycling); and (IV) cultural (including recreational and spiritual benefits) (Millenium Ecosystem Assessment, 2005). Ecosystem services are crucial for our well‐being and survival, highlighting the importance of preserving and sustainably managing our natural environments.

**Fig. 1:**
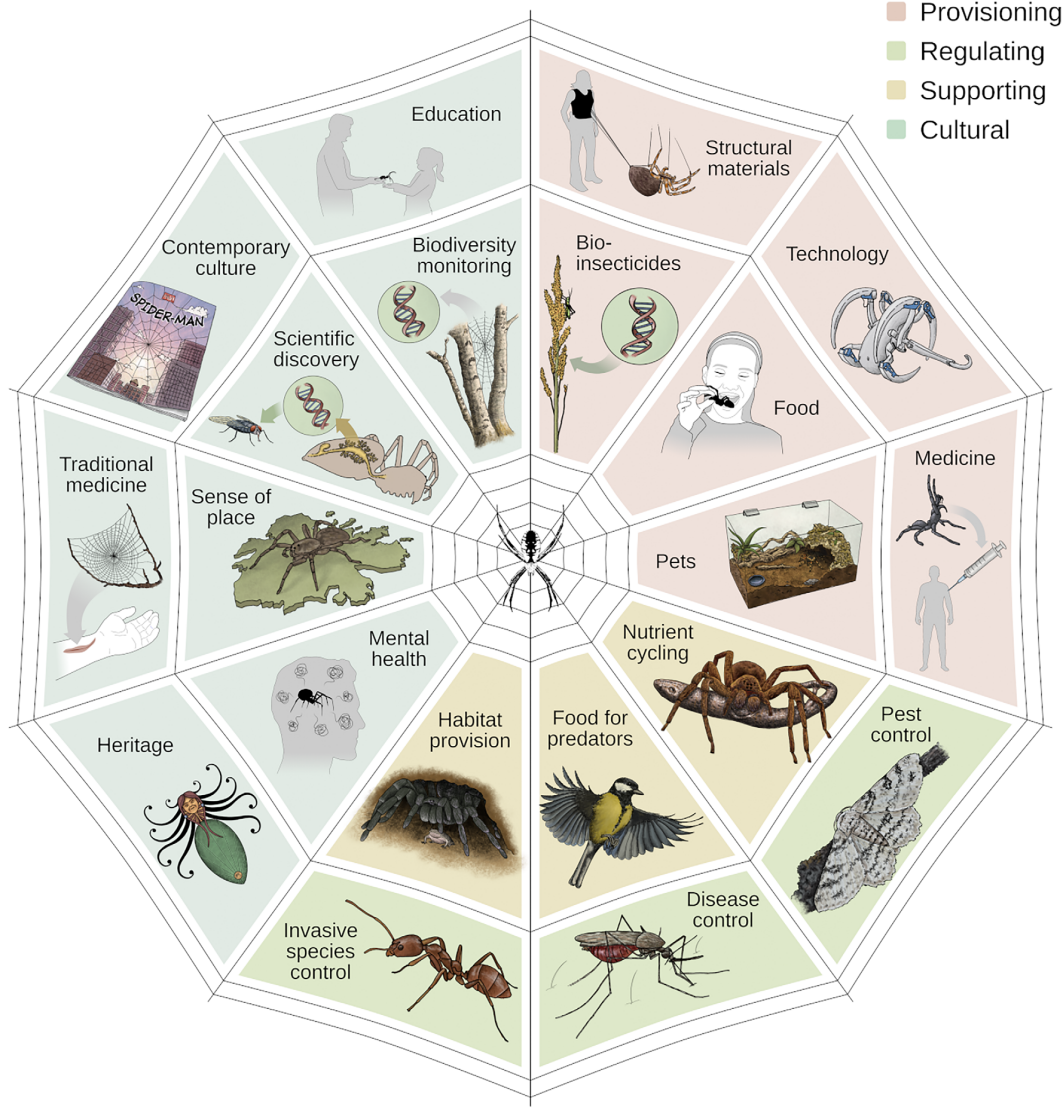
Ecosystem services provided by spiders. These services are categorised into four main types: (I) Provisioning; (II) Regulating; (III) Supporting; and (IV) Cultural.

Although critical ecosystem services are already described for many groups, most notably bees and other pollinators among invertebrates (Vanbergen & the Insect Pollinators Initiative, 2013; Requier *et al*., 2023), both the recognition and funding for other equally important taxa that provide critical services are still lacking. Even the ecosystem service literature often overlooks spiders. Following the same trend, nature‐based solutions provided by spiders have mostly been ignored. Nature‐based solutions use the inherent functions of ecosystems to address societal and environmental challenges (Cohen‐Shacham *et al*. 2016; Dunlop *et al*. 2024). By using what nature already provides, nature‐based solutions can help us solve biodiversity erosion, climate change and societal needs by restoring, preserving, or mimicking natural processes to enhance or provide ecosystem services. Here we summarise the many ecosystem services provided by spiders (Fig. 1) and how they can provide or inspire nature‐based solutions. We highlight numerous cases that illustrate their services and share new insights and our views on the future of this research area.

## II. PROVISIONING SERVICES

Provisioning services are those that humans directly and materially benefit from. They include structural materials, engineering solutions, medicine, insecticides, food, and pets.

### (1) Structural materials

The ability to produce silk and webs is probably the most distinctive feature of spiders. Spiders use silk in a variety of ecological contexts, from prey capture to defence, locomotion, and reproduction. For these purposes, each spider possesses tens to hundreds of silk glands that can be distinguished into up to eight different types, each producing a secretion with distinct properties (Blackledge & Hayashi, 2006). Both the versatility and sometimes outstanding mechanical properties of silk have prompted efforts to explore its potential applications, from textiles to biomedical materials (Kluge *et al*., 2008). Spider silk proteins (spidroins) are characterised by repetitive amino acid sequence motifs that result in a complex composite structure exhibiting mixed mechanical properties (Hayashi, Shipley & Lewis, 1999). For example, major ampullate spidroin 1 (MaSp1), one of the major spidroin compounds of dragline silk, exhibits alternating amorphous glycine‐rich domains and crystalline poly‐alanine beta sheets making it both strong and elastic. This combination makes it one of the toughest materials in nature (Agnarsson, Kuntner & Blackledge, 2010; Vollrath, Porter & Holland, 2011).

Because it is challenging to rear spiders on a large scale, the best approach is to mass‐produce spidroins using biotechnological methods (Blamires, 2024). They can then be processed into materials, such as fibres, films, and gels (Kluge *et al*., 2008; Koeppel & Holland, 2017). Replicating the properties of spider silk using such techniques, however, has proved difficult. There is still a limited understanding of the crucial structure–function relationships across the vast diversity of silk sequences and compositions and of the physical and chemical processes taking place during silk spinning (Wolff *et al*., 2017). The large size of natural spidroins also makes their synthesis difficult (Schmuck *et al*., 2023). However, recent efforts are nearing the goal of creating synthetic spider silks capable of exhibiting toughness values comparable to, or even exceeding, those of natural silks and of many artificial polymer materials (Anton *et al*., 2017). For example, scientists have recently used gene‐editing techniques to make silkworms produce spider silk, resulting in fibres that surpass the toughness of Kevlar (Mi *et al*., 2023). Another strategy for material innovation draws bioinspiration from spider silk, whereby the principle of alternating amorphous and crystalline domains is applied to engineered materials. This is another promising route for designing super‐tough materials (Gu, Jiang & Hu, 2019). Recently, the bioprospecting of spider silk was advanced by establishing the online *Silkome* database (https://spider-silkome.org/) with spidroin sequences from over 1000 species, and mechanical and structural properties of dragline silks for over 400 species (Arakawa *et al*., 2022). This rich resource permits the training of artificial intelligence (AI) tools to design new polymers with hitherto unachieved tensile performance (Lu, Kaplan & Buehler, 2024).

Natural spider silk contains not only fibre materials, but also adhesives (e.g. piriform and aggregate silk), and some research programmes have attempted to synthesise their compounds to produce sustainable adhesives (Liu *et al*., 2021). Notably, spiders rarely use single fibres and silk materials in isolation, but rather combine them into fabric‐like materials or architectures, such as threads, sheets, or webs (Liprandi *et al*., 2024). Such meta‐materials further increase the diversity of silk material properties and functions and have a hereto barely tapped potential for a wide range of biomimetic and bioinspired applications. For example, the knitting of knotted structures creates a tough meta‐structure (Koebley, Vollrath & Schlepp, 2017; Liprandi *et al*., 2024), and the combination of comparably stiff and strong threads with highly extensible threads in orb webs results in a structure that can sustain high kinematic impacts with minimal material consumption, and could inspire new safety devices and light‐weight constructions (Qin *et al*., 2015).

Using genetically modified bacteria to produce the fundamental building proteins of silk is being tested at reasonable scales and shows promise for the future (Bhattacharyya *et al*., 2021). So far, spider silk research has been mainly focused on the dragline silk of orb weaving spiders, but different spiders are able to produce different silk types with contrasting properties, meaning that there is still a large field to explore and optimise. We anticipate that genetic engineering of silk and the development of new chimeric spider silks with enhanced functions and specific characteristics will reveal the biomimetic potential of spiders through expressed spider‐silk proteins, in the control of self‐assembly processes and the selective exploration of material applications (Kluge *et al*., 2008).

Beyond silks, the spider's cuticle also provides a powerful source of inspiration for high‐performance structural materials. Through differences in the structural arrangement of chitin fibrils or the inclusion of metal ions, an enormous range of mechanical properties can be achieved from the same basic compounds (Politi *et al*., 2021).

### (2) Engineering and technology

**Fig. 2:**
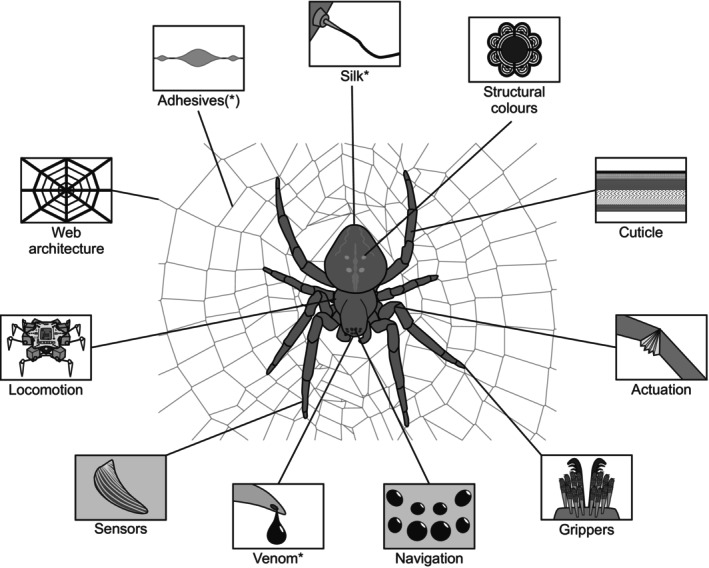
Provisioning services: bioinspiration and bioprospecting. Spiders are a rich source for the discovery and isolation of new materials, drugs and bioactive compounds (bioprospecting) or working principles (structure–function relationships; biomimetics and bioinspiration) that can be used in or inspire the development of new technologies and applications. Items marked with an asterisk are used directly, e.g. extraction of compounds for direct use or as blueprints for biotechnological synthesis of functional compounds; brackets indicate that the item is equally important in bioprospecting and bioinspiration.

Not only has spider silk generated interest in terms of biomimetics, but the unique locomotor and sensory adaptations of spiders have already inspired technologies and engineering products (Fig. 2). Their use of hydraulic pressure to extend their limbs particularly inspires engineers (Landkammer *et al*., 2016). In the field of robotics, prototypes have been developed that mimic the octopodal walking pattern of spiders (Vidoni & Gasparetto, 2011), as well as dragline abseiling behaviour (Wang, Culha & Iida, 2014). In addition, the remarkable locomotory behaviour of the Moroccan flic‐flac spider *Cebrennus rechenbergi* Jäger, 2014, a desert‐dwelling species capable of rolling down dunes at high speeds (estimated at 2 m/s), has served as an inspiration for the construction of a robot with similar locomotory elements suitable for sandy or snowy terrain (King, 2013).

The structural colour of some spiders has inspired the development of new structural dyes that can be used as pigment substitutes in materials such as plastics, metals, textiles, and paper, and to produce colour for wide‐angle viewing systems such as phones and other electronic devices (Hsiung *et al*., 2017*a*,*b*). The structural principle of hairy adhesive foot pads (claw tufts) of many cursorial spiders, such as jumping spiders, which provide rapid reversible adhesion without glues, could inspire smart adhesives for robotics and the handling of fragile items in assembly lines (Seidl & Vidoni, 2012).

In the field of acoustics, new sensors based on nanoscale crack junctions inspired by the geometry of a spider's slit organ have been demonstrated to achieve ultra‐high sensitivity, allowing them to serve multiple purposes (e.g. microphones; Kang *et al*., 2014). Similarly, the ability of a desert‐dwelling spider species (*Leucorchestris arenicola* Lawrence, 1962) to move at night without cues from the sun, air‐borne chemicals, wind direction, or vibration (Nørgaard, 2005) could inspire new navigation devices for environments that lack these cues (e.g. for autonomous motor vehicles designed to travel on the surface of celestial bodies).

### (3) Medicine and health

Spider venom, hemolymph and highly specialised types of silk exhibit a range of bioactive properties that are currently being assessed in bioprospecting programmes to identify new candidate compounds for advanced therapeutics for a number of diseases, some of which are currently hard to treat.

Venom of a single spider species usually comprises a hundred to a thousand components (Chassagnon *et al*., 2017). Within their staggering species diversity, spider venoms represent a huge combinatorial library of millions of biochemicals. The main compounds of spider venoms are disulfide‐rich neurotoxins that target a variety of receptors and ion channels with different levels of specificity (Escoubas, Diochot & Corzo, 2000; Vassilevski, Kozlov & Grishin, 2009; Kuhn‐Nentwig, Stöcklin & Nentwig, 2011). Structurally, most spider neurotoxins contain an inhibitor cystine knot (ICK) or similar fold, which provides exceptional stability to the toxin molecule (Herzing & King, 2015). Due to these properties, spider venom peptides have potential therapeutic applications (Saez & Herzig, 2019).

Some spider neurotoxins show potential for therapeutic applications to treat neurodegenerative diseases, pain, skeletal muscle diseases, skin disorders, urinary system disorders, metabolic disorders, cancer, vascular disorders, microbial infections, or malaria, and exhibit analgesic and neuroprotective properties (Akef, 2018; Saez & Herzig, 2019). Research on the neurodegeneration mechanisms of *Cupiennius salei* (Keyserling, 1877) venom led to a groundbreaking discovery that sheds light on how Alzheimer's disease develops in the human brain (Fabian‐Fine *et al*., 2024) Currently, the most promising drug candidate comes from the venom of an Australian funnel‐web spider [*Hadronyche infensa* (Hickman, 1964)] (Chassagnon *et al*., 2017). The venom peptide Hi1a targets acid‐sensing ion channel 1a (ASIC1a), which plays a crucial role during ischemic stroke and cardiac arrest (Chassagnon *et al*., 2017). Hi1a has cardioprotective properties against myocardial ischemia and improves the viability of donor hearts for transplantations (Redd *et al*., 2021). The compound is currently progressing towards commercial drug development.

Spider hemolymph has also been found to contain peptides with antimicrobial activity, which play a role in their immune defence against pathogens. These peptides have shown potential applications in medicine, particularly as novel agents for combating antibiotic‐resistant bacteria, fungi, and multiple infectious diseases (Riciluca *et al*., 2012; Sangavi *et al*., 2023).

Some spider silks are also biodegradable and biocompatible, i.e. are invisible to the human immune system. This combination of traits renders spider silk very interesting as a biomaterial for advanced biomedical technologies. For example, spider silk can be used to encapsulate drugs and deliver them to targeted tissues (Leal‐Egaña & Scheibel, 2010; Omenetto & Kaplan, 2010). Other potential medical applications include engineering artificial silk that breaks down in the body after a certain period, allowing it to be used in internal surgery without the need to remove stitches after healing (Omenetto & Kaplan, 2010). Spider silk also provides excellent cell adhesion properties useful for tissue engineering and artificial nerve construction, where the silk acts as a scaffold between two severed nerves to promote faster and more efficient nerve regeneration (Schacht & Scheibel, 2014; Semmler *et al*., 2023).

Spider venoms, hemolymph and silk hold promise as a library of compounds for therapeutics that has countless possibilities (Saez & Herzig, 2019). New advances in using machine learning for the discovery of new drugs (Dara *et al*., 2022) might radically speed up the screening of candidates for multiple uses, as well as the development of ways to produce new compounds efficiently.

### (4) Bio‐insecticides

Almost all spiders use venom to paralyse their prey (Kuhn‐Nentwig *et al*., 2011). As insects are the main prey of spiders (Nyffeler & Birkhofer, 2017), many spider venom peptides are neurotoxins with insecticidal properties that target the insect nervous system. Also, spider venoms are highly stable in the insect gut and hemolymph, making them ideal candidates for the development of highly specific eco‐friendly insecticides. Transgenes encoding venom peptides can be used to develop insect‐resistant crops or enhanced entomopathogens (King & Hardy, 2013). There is currently one commercial biopesticide based on the spider neurotoxin GS‐omega/kappa‐Hxtx‐Hv1a peptide from the Blue Mountains funnel‐web spider *Hadronyche versuta* (Rainbow, 1914). It targets greenhouse pests such as thrips, spider mites, whiteflies, and aphids, but does not affect mammals or honeybees (Bomgardner, 2017; King, 2019). Several other spider toxins have been targeted as potential candidates for bioinsecticides based on criteria such as potency, selectivity, oral toxicity, bioavailability, stability, and the cost of large‐scale production (Saez & Herzig, 2019).

Potential synthetic production of venom‐like insecticides still presents multiple avenues for future exploration (King & Hardy, 2013). There is much potential in studying spider venom‐like insecticides using similar approaches to those used for drug discovery (Dara *et al*., 2022). The main issue from an ecological perspective will be selectivity, as an ideal eco‐friendly insecticide should be selective towards target pests but harmless for other arthropods. Therefore, spider toxins with a higher selectivity towards one arthropod taxon should be investigated in more detail (Michálek, Kuhn‐Nentwig & Pekár, 2019).

### (5) Food

Spiders are traditional food sources across Asia (e.g. Thailand, Cambodia, China), Oceania (e.g. Papua New Guinea, New Caledonia), Africa (e.g. South Africa, Gambia, Madagascar), and the Americas (e.g. Mexico, Paraguay, Venezuela) (Costa‐Neto & Grabowski, 2021). The most famous example is in Cambodia, where the small town of Skun is known to locals as ‘spider town’, due to its practice of selling fried tarantulas to visitors. Called *a‐ping* by Cambodians and most likely from the genus *Haplopelma* (Theraphosidae), these tarantulas were traditionally consumed for medicinal practices. The shift to treating them as food sources is thought to have occurred more recently, due to widespread starvation under Pol Pot's Khmer Rouge regime in the 1970s. Other Theraphosidae and the sparassid *Heteropoda venatoria* (Linnaeus, 1767) are an important part of the diet of Yanomami Indigenous communities in Venezuela (Costa‐Neto & Grabowski, 2021). They are collected from the wild and typically prepared by heating, defanging and, in certain cases, drying and grinding. The Edible Insects Program at the Food and Agriculture Organization (FAO) has examined the potential of spiders as human food (Costa‐Neto & Grabowski, 2021). However, it should be noted that with the impracticalities of mass rearing, using spiders as a substantial food source is currently unsustainable at large scales.

### (6) Pets

People around the world keep spiders as pets. Many large tarantulas (Theraphosidae) have long lifespans, sometimes reaching several decades in captivity (Mason, Wardell‐Johnson & Main, 2018). The legal tarantula trade can be important for local livelihoods in some areas. In Mexico, for example, some breeders are allowed to breed native species and sell their captive‐bred offspring to national and international pet markets. The exact number of spider species kept as pets remains unknown and estimates of the volume of spiders in the pet trade are difficult to obtain (Marshall *et al*., 2022; Fukushima *et al*., 2023). The global popularity of pet spiders has been facilitated by the widespread availability of these arachnids through online platforms (Marshall *et al*., 2022). This is particularly true for tarantulas, which garnered enthusiasm as pets in the 1970s, primarily in Europe and the USA, where dedicated hobbyist societies were formed. Since then, the hobby has grown exponentially worldwide (Marshall *et al*., 2022), with enthusiasts keeping hundreds of species of different genera. In some cases, desirable species such as *Typhochlaena seladonia* (C.L. Koch, 1841) can reach prices in excess of US$ 250 for a spiderling. Prices and availability of tarantulas in the pet trade are often influenced by species' traits, with hairy and aggressive tarantulas being more abundant in the pet market (Fukushima *et al*., 2023).

In addition to the well‐known theraphosid spiders, other mygalomorph spiders such as *Cyclocosmia* Ausserer 1874 and *Ummidia* Thorell 1875 (Halonoproctidae) and species from other families such as Theridiidae, Thomisidae, Sparassidae, Lycosidae, Ctenidae, Araneidae, and Liphistiidae are kept as pets (Marshall *et al*., 2022). Common garden‐dwelling species in the tropics, such as *Trichonephila* sp. and *Nephilingis cruentata* (Fabricius, 1775) are also sold and kept as pets. Jumping spiders (Salticidae, e.g. *Phidippus* spp.), known for their cognitive abilities and capacity to interact with visual stimuli from humans, as well as velvet spiders (Eresidae, e.g. *Eresus* spp.), have also recently gained popularity.

Captive breeding and rearing, when complying with legal, ethical, scientific, and sustainable practices, may be a useful conservation tool (Fukushima *et al*., 2021). For some spider species, it could help to reduce poaching to meet the pet market demand; however, additional studies are required to understand the implications of these practices fully. Furthermore, species in captivity can serve as reservoirs in case of losses or extinction of wild populations. For example, the Desertas wolf spider (*Hogna ingens*), a species present in a single valley on Madeira Island, Portugal (Crespo *et al*., 2014), is part of the *ex situ* programme of the European Association of Zoos and Aquaria and is bred in zoos across Europe to ensure population restoration in case of ecosystem loss (Bushell *et al*., 2018). Additionally, captive individuals can be used for educational purposes, improving knowledge and raising awareness of spiders, their importance, and their conservation issues to the general public. In Mexico, for example, some Units for Management and Sustainable Exploitation of Wildlife [Unidades de Manejo y Aprovechamiento Sustentable de Vida Silvestre (UMA)] maintain and breed tarantula spiders for reintroduction, exhibition and education, but also for sustainable commercial use, improving the livelihood of the local community (CEC, 2017).

## III. REGULATING SERVICES

Regulating services are those that contribute to ecosystem stability. For spiders, these include, in decreasing order of evidence available, pest control, disease control, and invasive species control.

### (1) Pest control

The great majority of spider species are generalists that prey on other arthropods. Although there is no available evidence that any spider species alone, or in a community of other spiders, can control pests, there is a body of evidence showing marked pest population suppression; although for a short period of time and under specific conditions (Michalko *et al*., 2019*a*). This absence of evidence for targeted biocontrol is because spiders lack some attributes of a successful biocontrol agent. Although they have a high functional response (including overkilling), their numerical response is often insufficient, they are rarely pest specific, and they have slow ontogenetic development compared to that of target pests (Michalko, Pekár & Entling, 2019*b*). Their pest suppression potential is further compromised by their frequent predation on other beneficial arthropods (other predators and pollinators). Consequently, while predominantly inefficient target biocontrol agents, spiders are nonetheless an essential part of conservation biological control, which is based on supporting their natural abundance through the management of habitats as part of preserving the natural enemy community.

So far, only 11 studies have investigated the impact of spiders on pest populations in perennial systems through manipulative experiments, nine of which found that spiders significantly reduced pest densities, including moths, psyllids, coccids, and planthoppers (Michalko *et al*., 2019*a*). Perennial crops, such as orchards, horticultural plantations, and vineyards, are relatively stable agroecosystems in comparison to annual and greenhouse crops and they usually host abundant and diverse spider communities (e.g. Isaia *et al*., 2010; Happe *et al*., 2019). The few existing studies using manipulative experiments indicate that spiders are effective in pest suppression in perennial crops. Studies investigating whether the effect of spiders on pests affects crop production are rare but show promising findings. For example, Isaia *et al*. (2010) increased spider densities by installing refuges in the form of corrugated cardboard bands around apple trees. The increased number of spiders led to a reduced number of moth pests and increased apple production. Similarly, the installation of corrugated cardboard on pear trees led to an increased number of spiders, a reduction of psyllid pests, and an increase in pear production (Michalko *et al*., 2017). In the temperate zone, many arthropods overwinter on fruit trees (Michalko *et al*., 2022). Some spiders remain active during winter and can prey on pests even at sub‐zero temperatures (Pekár *et al*., 2015; Gajski *et al*., 2024). Indeed, winter‐active spiders are highly effective at pest suppression in pear orchards during the non‐growing season (Pekár *et al*., 2015; Michalko *et al*., 2017, 2022; Gajski *et al*., 2024), a crucial period that may determine the trajectories of pest population growth.

Experimental studies indicate that spiders contribute to pest suppression in annual crop fields too (e.g. Lang, Filser & Henschel, 1999; Öberg, Cassel‐Lundhagen & Ekbom, 2011; Holland *et al*., 2012), and may even improve crop plant performance (Snyder & Wise, 1999; Oelbermann & Scheu, 2009). A global synthesis shows that agrobiont spiders have significantly higher proportions of aphid prey in their diets compared to species in non‐agricultural habitats (Birkhofer *et al*., 2022). Initial cereal aphid densities were reduced by 50% through actively hunting spiders alone as compared to spider‐free controls or treatments with only web‐building species. This aphid suppression, however, was not persistent over time and did not provide sufficient control to avoid subsequent rapid aphid population growth (Birkhofer *et al*., 2008). Nevertheless, higher levels of aphid predation by web‐building spiders and lower aphid numbers can still be associated with higher cereal yields (Birkhofer *et al*., 2016).

Finally, spider biocontrol potential in greenhouses is the least studied in comparison to perennial and annual agroecosystems, yet offers many future research possibilities. As spider assemblages need to be mass collected and introduced into greenhouses to provide biocontrol services, and because different plant species harbour different spider communities (e.g. Bán, Fetykó & Tóth, 2012), further studies should investigate what vegetation types provide a suitable arthropod composition for introduction in greenhouses for a given crop type as well as how spider presence and diversity might influence crop yields. Finally, assessments of the importance of spiders in forest ecosystems are lacking, despite the economic importance of forest products and abundance of forest insect pests.

Further investigations are needed using manipulative studies in the Americas, Africa and mainland Asia (Michalko *et al*., 2019*a*), especially in tropical agroecosystems for which information is scarce. Despite some progress, gaps in understanding persist, particularly regarding the biocontrol function of spiders in non‐perennial crops and in any crops of tropical agroecosystems across the globe.

### (2) Disease control

In their role as common and widespread arthropod predators, spiders can prey on species relevant to human health. Arthropod vectors of human disease include dipterans (mosquitoes, sand flies), hemipterans (true bugs), fleas, lice, ticks, and trombiculid mites (Weitzel *et al*., 2020). These vectors transmit malaria, leishmaniasis, typhus, Chagas disease, Lyme borreliosis, and encephalitis, for example. Across these vectors, spiders are known to prey on dipterans, hemipterans, and ticks (Nentwig, 1987, Burtis & Pflueger, 2017). Fleas, lice, and trombiculid mites have not been reported as prey of spiders, presumably due to their strong host (human body) association. By preying on vectors, spiders may, to a certain extent, reduce pathogen prevalence and rates of disease transmission.

The potential for spiders to reduce arthropod vector prevalence depends in part on whether the vectors occur indoors or outdoors. Limited immigration rates result in lower population densities of vectors indoors, making a reduction of such populations achievable by the nature‐based solutions approach, i.e. support of naturally occurring indoor spiders. Many synanthropic indoor spider species, both cursorial and web‐building, capture dipterans or reduviid bugs and thus could be effective for indoor vector control. Indeed, pholcids and salticids are known to have a high functional response to mosquitoes under laboratory conditions (Ndava, Llera & Manyanga, 2018). The best‐known example of an indoor bioagent, however, is a social species, *Mallos gregalis* (Simon, 1909) (Dictynidae), called *el mosquero* in Mexico. The webs of *el mosquero* capture many flies due to an attractive odour (Tietjen, Ayyagari & Uetz, 1987). Evidence of vector‐specific foraging comes from the salticid *Evarcha culicivora* Wesołowska & Jackson, 2003 from East Africa, which was found preferentially to select *Anopheles* mosquitoes and in particular females engorged with blood (Nelson & Jackson, 2006).

Due in large part to the availability of multiple taxa of prey in natural environments, it is less likely that spiders can significantly reduce populations of vectors that mainly occur outdoors (sand‐flies, mosquitoes, and ticks). Nonetheless, spiders are known to consume mosquito larvae and emerging adults in aquatic habitats. Additionally, sand‐flies and ticks fall prey to both cursorial and web‐building spiders, but at a relatively low frequency (Samish & Alekseev, 2001). For a lycosid, *Schizocosa ocreata* (Hentz, 1844), in North America, a field experiment determined that its presence and cues left on surfaces markedly decreased the abundance of ticks locally (Burtis & Pflueger, 2017).

### (3) Invasive species control

Predator–prey interactions within both natural and anthropogenic ecosystems often have implications for the management of invasive species. While we could not find examples of intentional use of spiders to control invasive species, observations of incidental interactions and targeted predation by spiders shed light on their potential role in this context. For instance, araneid spiders construct non‐selective webs that capture various prey, including invasive species like *Harmonia axyridis* ladybugs (Sloggett, 2010). Specific predatory behaviour by certain spider species also highlights a more direct influence on invasive species populations. Ant‐eating *Zodarion* spiders, for example, were observed predominantly hunting *Linepithema* ants in Portugal (Pekár *et al*., 2018). These ants are aggressive invasives that often disrupt trophic networks with implications for the conservation and extinction risk of other insects.

## IV. SUPPORTING SERVICES

Supporting services are those that allow other services to function normally. These include nutrient cycling, serving as food for predators and habitat provision for other organisms.

### (1) Nutrient cycling

Nutrient cycling is one of the most important ecosystem services (Costanza *et al*., 1997) and spiders can affect nutrient cycling directly and indirectly (Schmitz, Hawlena & Trussell, 2010). The direct ways include excretion, defecation, and disposal of prey remains (Herzog *et al*., 2023; Wilder *et al*. 2025). The excreta and the disposed prey remains of the Neotropical jumping spider *Psecas chapoda* (G. W. Peckham & E. G. Peckham, 1894) – a species that is strictly confined to terrestrial bromeliads – contributes 18% of the nitrogen requirements of the host plant. Moreover, the symbiosis between bromeliads and this spider results in a 15% increase in leaf length in comparison to bromeliads without the spider resident (Romero *et al*., 2006). Linyphiid spiders are often the earliest colonists of newly exposed substrates on glacier forelands, due to their airborne dispersal in the form of ballooning. These spiders capture passing insects and the discharged prey remains probably represent an important allochthonous resource of nitrogen and phosphorus for the developing ecosystem at the earliest stages of succession (Hodkinson *et al*., 2001). In a mesocosms experiment, Wilder *et al*. (2025) showed that the black widow spider *Latrodectus mactans* (Fabricius, 1775) and the Carolina wolf spider *Hogna carolinensis* (Walckenaer, 1805) enriched soil through the deposition of their waste products (faeces and prey remnants) that consequently led to enhanced growth and biomass of *Brassica* plants.

Spiders can also influence nutrient cycling indirectly by triggering trophic cascades in detritus‐based food chains. They have been shown significantly to slow down, as well as to accelerate, litter decomposition in forest floors. The direction of trophic cascades depends, in part, on both humidity and spider hunting strategy (Lawrence & Wise, 2000, 2004; Lensing & Wise, 2006). Liu *et al*. (2015) observed that in a tropical forest under ambient moisture, cursorial spiders but not web‐building spiders enhanced litter decomposition, most likely by predation on natural enemies of oribatid mites, which are important decomposers. Under drought conditions, however, both web‐building and cursorial spiders slowed the decomposition rate, probably because of predation on entomobryid collembola, which were the primary decomposers.

Spiders can influence nutrient cycling by triggering trophic cascades not only in the detritus‐based food‐chain but also in the plant‐based food‐chain because they influence the quality and quantity of organic matter entering the decomposition and mineralisation processes. A series of studies have demonstrated that spiders significantly influence the cycling of carbon and nitrogen in a grassland ecosystem. For example, Schmitz (2008) observed that the actively hunting spider *Phidippus rimato*r (Walckenaer, 1837) indirectly caused a reduction in plant species evenness and enhanced aboveground net primary production and nitrogen mineralisation. By contrast, the sit‐and‐wait spider *Pisaurina mira* (Walckenaer, 1837) had positive effects on plant species evenness but reduced aboveground net primary production and nitrogen mineralisation. Nitrogen mineralisation in the actively hunting spider treatments was 33% higher than in sit‐and‐wait spider treatments (Schmitz, 2008). The presence of the sit‐and‐wait spider *P. mira* reduced herbivory by the grasshopper *Melanoplus femurrubrum* (DeGeer, 1773), leading to increased ecosystem‐level carbon retention because plants reduced respiration and allocated more carbon to their belowground parts (Strickland *et al*., 2013). Moreover, Hawlena *et al*. (2012) showed that the mere presence of *P. mira* indirectly affects soil community function, apparently *via* the amount of herbivore protein entering the soil.

### (2) Food for other organisms

Spiders are consumed by a wide range of predators, including birds, mammals, amphibians, reptiles, fishes, and other arthropods, contributing to the trophic structure of ecosystems. Spiders are often an important food source for birds, particularly in forest ecosystems (Gunnarsson, 2008). Spiders comprise more than 60% of prey items consumed by goldcrests (*Regulus regulus*) in winter (Hogstad, 1984) and up to 75% of biomass fed to great tit (*Parus major*) nestlings early in the breeding season (Naef‐Daenzer, Naef‐Daenzer & Nager, 2000). Predation by lizards and frogs can decrease spider abundance on islands (Schoener & Toft, 1983; Beard *et al*., 2021) and spiders are among the most common prey (by volume) in the diet of rainforest frogs (Luría‐Manzano & Ramírez‐Bautista, 2017). Spiders also contribute to the diets of mammals including bats and monkeys (e.g. Fonseca *et al*., 2022). Despite being primarily terrestrial, spiders can also be prey for marine and freshwater fishes (Chan, Zhang & Dudgeon, 2008; Bollens *et al*., 2010). Among invertebrates, spiders have been reported to be prey of other Arachnida, Platyhelminthes, Pauropoda, Chilopoda, and insects (Pekár & Raspotnig, 2022). Few predators are specialised on spiders, but araneophagous spiders can be found in Archaeidae, Caponiidae, Gnaphosidae, Lamponidae, Mimetidae, Palpimanidae, and Salticidae (Pekár & Toft, 2015). Several assassin bugs (Reduviidae) are also specialised on web‐building or social spiders and utilise them throughout their development (Jackson, Salm & Nelson, 2010).

Parasitic organisms that use spiders as hosts include microparasites (viruses, bacteria, fungi) and macroparasites (invertebrates). Many spiders seem to be hosts of RNA or DNA viruses, but evidence is rather scarce (Durkin *et al*., 2021). Spider bodies are often infected by extracellular pathogenic or intracellular endosymbiontic bacteria which do not seem to be specialised on spiders (Goodacre, 2011). Bacteria, such as *Wolbachia* or *Cardinium*, are transmitted among spiders both horizontally and vertically and are known to manipulate the behaviour of their hosts (Durkin *et al*., 2021). Besides bacteria, fungi (both pathogenic and non‐lethal species) use entire spider bodies to develop mycelia in humid habitats, infecting a high diversity of spider species. In addition to these microparasitic organisms, nematodes are endoparasitic on spiders. Another group of macroparasites are mites from many different families, and these are typically ectoparasites (Durkin *et al*., 2021).

Parasitoids of spiders are mostly insects, which kill the host prior to completion of their development. Parasitoids use either eggs or nymphs of spiders to feed their developing offspring. Egg parasitoids include numerous families of wasps, lacewings, flies, and moths (Austin, 1985) and they typically consume the whole egg clutch. There are many ectoparasitoids of spider nymphs, particularly among Pompilidae and Ichneumonidae wasps (Fitton, Shaw & Austin, 1987) and flies (Gillung & Borkent, 2017). While many hymenopteran parasitoids are highly specialised on spiders, dipteran parasitoids also attack insects.

### (3) Habitat provision

In a commensal relationship, spiders provide habitat for other organisms like yeasts (Tietjen *et al*., 1987) and mites (Bernardi *et al*., 2017). These organisms cohabit in the webs and retreats of dictynid, agelenid, and hexathelid spiders throughout their lifespan. Yeasts, for instance, thrive on prey carcasses ensnared in the web, attracting flies beneficial for *Mallos* spiders. The precise role of mites in spider webs remains unclear due to incomplete information.

Reports on the utilisation of spider retreats are scarce. Some burrowing spider species like *Xenesthis*, *Haploclastus*, and *Poecilotheria* have been observed sharing their retreats with frogs (Cocroft & Hambler, 1989, Siliwal & Ravichandran, 2008). In this association, frogs find shelter and can feed on decaying prey remnants of the spider. The nests of the social velvet spider *Stegodyphus sarasinorum* Karsch, 1892 provide shelter for a range of commensals, from insects to small vertebrates (Jani *et al*., 2023). Additionally, abandoned igloo‐shaped retreats of *Zodarion* spiders often house soil‐dwelling arthropods like pseudoscorpions and mites (S. Pekár, unpublished data). Spider webs are also used as construction material for nest building by small birds (Low *et al*., 2013).

Koinobiont parasitoids, which allow their host to grow and develop, not only consume the spider body but also use the webs of host spiders during pupation. The web offers protection to the parasitoid pupa from predators as well as unfavourable conditions (Korenko *et al*., 2022). Prior to parasitoid pupation, the original web design of the host spider is often altered due to manipulation by the parasitoid larva (Eberhard, 2000).

Spider webs often contain prey that are exploited by other organisms. Kleptobionts from various taxonomic groups have been observed in spider webs, including other spiders, mites, dipterans, lepidopterans, hemipterans, hover wasps, scorpionflies, dragonflies, and birds. While the cohabitation of kleptobionts is often seen as parasitism or commensalism, experimental evidence suggests it might also be mutualistic. For example, *Psechrus* webs hosted by *Philoponella* spiders attract more prey and removing *Philoponella* led to a decreased growth rate of *Psechrus*, indicating a potential mutualistic relationship between the two spider species (Elgar, 1994).

## V. CULTURAL SERVICES

Cultural services are those that provide us with a sense of home and well‐being, aesthetic inspiration, cultural identity, or allow scientific endeavour. Spiders are known by all, frequently encountered throughout our lives, and have the uncommon ability to evoke both fear and fascination. It is thus no surprise that spiders are omnipresent in human culture and a subject of intense scientific investigation, making them significant provisioners of cultural services.

### (1) Spiritual and cultural heritage

Spiders have served as both symbols of creativity and fear, weaving their way into our stories, art and collective imagination. Uttus, the ancient Sumerian goddess of weaving, was envisioned as a spider spinning her web. Anansi is a prominent figure in the folklore of West Africa, particularly among the Ashanti people of Ghana. Anansi's stories, passed down through generations, serve as a vehicle for teaching moral lessons and conveying cultural values. He represents wisdom and how small creatures can outsmart larger ones, often using the seemingly complex and intelligent building of a web. In Native American folklore, Spider Woman is often depicted as a weaver, responsible for spinning the web of life, symbolising the interconnectedness of all living beings. In Japan, the Tsuchigumo and Jorogumo are spider creatures that can transform into a woman. In Greek mythology, Arachne's myth serves as a cautionary tale about the consequences of human pride, emphasising the connection between creativity and the spider.

Spiders have also inspired dance as a form of healing. The Italian dance ‘Tarantella’, with roots from the 15th century, is said to be caused by the bite of the wolf spider *Lycosa tarantula* (Linnaeus, 1758). The victim would be overtaken by contortions, dancing convulsively or entering a state of delirium. They would continue dancing until exhaustion, at which point they were considered cured.

‘Arachnomancy’ or divination by interpreting spiders' appearance, behaviour, or the movement of their webs is observed in different countries. A spider's apparent forethought in preparing the web to capture future prey might be perceived as predicting the future. In European druidic tradition, spiders were considered as a balance between the past and the future through the construction of the web, with the spider showing that we must decide our future.

Within mainstream religion, spiders hold special significance in Islam, as they are widely believed to have saved the life of Muhammad when a spider spun a web to cover the entrance of a cave, concealing the prophet from his enemies (Nadeem & Nadeem, 2022). A similar incident is reported in the Torah, this time with David as protagonist (Nadeem & Nadeem, 2022). The spider web is mentioned both in the Quran and the Bible to illustrate the fragility of confidence of non‐believers.

The revival of some of these old, often forgotten, traditions and/or stories might be a novel approach to change the current dominant perception of spiders as synonymous with danger. In many ancient cultures, spiders were admired as capable of complex behaviour and they often symbolised interconnectedness. These positive portrayals should be reintroduced as important cultural services provided by spiders throughout the world. Balancing the values and costs of spiders to society aids in refining conservation narratives and agendas. To shape effective conservation strategies better, we must fully understand the complex relationships between societal perceptions, media influence, and human–spider interactions.

### (2) Traditional medicine

Humans across the world have used tarantulas as a treatment to cure respiratory problems (Costa Neto & Resende, 2004) or for skin and tooth problems (Costa‐Neto, 2006). The Tzetzales and Tzotziles ethnic groups in Chiapas, Mexico, use *Tliltocatl vagans* (Ausserer, 1875) to treat tumours by making a spider bite the affected area (Enríquez Vázquez *et al*., 2006). Likewise, Chol communities apply a solution of *T. vagans* juice to eyes to ‘clean the eyesight’ (Henaut, Tchibozo & Machkour‐M'Rabet, 2016). Tarantulas of the genus *Phoneyusa* are captured and fried for consumption to cure stomach ailments by the Senufo people in Mali (Henaut *et al*., 2016) and in Cambodia, the consumption of fried tarantulas (*Haplopelma* sp.) is also considered beneficial for respiratory problems.

Other spiders or their silk have also been used in traditional medicine. For example, spider webs are used by the Sukuma Tribe in Tanzania as bandages to treat wounds (Vats & Thomas, 2015). Crab spiders were used in India for healing the heart, throat, lungs, and back pain (Lev, 2006). Also in India, the huntsman spider *Heteropoda venatoria* (Linnaeus, 1767) is commonly used by the Char Chapori people for treatment of skin rashes, asthma, ulcer, and menorrhagia (Ahmed *et al*., 2013). Spiders were used in traditional medicine during the 17th to 19th centuries in England. If prepared or contained in certain ways, such as placed in a bag around one's neck, spiders were thought to help cure multiple maladies. Mashed spiders taken in pill form were even prescribed for malaria (Bristowe, 1958) and cobwebs were thought to stem bleeding and curb fevers (Jackson, 1809).

While these traditional practices hold cultural significance for local populations, their ecological impacts and medical efficacy are not well understood due to a lack of scientific research. Gaining deeper insight into these practices will be critical for designing conservation interventions that both respect cultural traditions and ensure ecological sustainability. Such understanding could also open opportunities for bioprospecting, provided it adheres to sustainable practices and benefit‐sharing agreements that fairly compensate the countries and communities that host these species. Interestingly, the use of spiders in traditional medicine can lead to positive conservation outcomes. For example, in the Maya Chol and certain Brazilian villages, tarantulas are positively perceived and their populations are protected in communities where they are used in traditional medicine (Costa‐Neto, 2006; Machkour‐M'Rabet, Rojo & Hénaut, 2011). By contrast, in areas where they hold no medicinal value, tarantulas are often viewed negatively and systematically eliminated.

### (3) Contemporary culture

Spiders have been a source of inspiration in various literary works and films. Tokien's Ungoliant and Shelob are giant spiders personifying darkness in *The Silmarillion* and *Lord of the Rings* books. In the *Harry Potter* series, Aragog was an ‘acromantula’ kept, when young, as a pet. The feature films *Arachnophobia* and *Eight Legged Freaks* depict fictitious spiders that wreak havoc in contemporary cities. Spiders are also often depicted positively. Eric Carle's *The Very Busy Spider* is one of the most successful early reading children's books in western countries. In E. B. White's *Charlotte's Web*, Charlotte the spider demonstrates the power of friendship and selflessness. Internationally acclaimed animator Hayao Miyazaki wrote and directed a short film named *Mizugumo Monmon* about a diving bell spider who falls in love with a water strider, a story about unlikely love and the beauty of aquatic life at the microscopic scale. Adrian Tchaikovsky won both Arthur C. Clarke and Hugo Awards for the science fiction series *Children of Time*, which features protagonists based on the remarkably intelligent jumping spider *Portia labiata* (Thorell, 1887). Marvel Comics' *Spider‐Man* is a classic coming‐of‐age narrative, and his arachnid‐inspired abilities symbolise the idea that even the most ordinary individuals can become heroes. According to Da‐Silva *et al*. (2014), there are at least 123 characters in comics other than Spider‐Man inspired by arachnids. In the internet era, *Lucas The Spider* is a popular cartoon on YouTube about a gentle, curious, and warm‐hearted jumping spider.

Spiders have also made their way into the world of music, from *Spiders* by System of a Down, *Tarantula* by The Smashing Pumpkins, or *As Aranhas* by Mão Morta, among others, often depicting themes of complexity, interconnectedness, or desolation. The nursery song *Itsy Bitsy Spider* is famous in anglophone countries and translated to many other languages. Spiders are also commonly represented in video games, from *Resident Evil* and *Skyrim* to *Zelda* and *Minecraft*, as dangerous creatures to overcome. In the *Pokémon* franchise, there are at least four different spider‐inspired bug‐type Pokémon and their associated evolved forms. The card game *Magic: The Gathering* includes 69 spiders within its characters (Milne & Derkarabetian, 2025).

Finally, contemporary art provides a wide range of spider‐related artworks in different forms and mediums. The iconic sculpture *Maman*, by the French‐American artist Louise Bourgeois, is a massive, 30‐foot‐tall spider made of bronze, stainless steel, and marble. Some of the work of the Argentinian artist Tomás Saraceno is centred on spiders. For example, in *Becoming Spider*, the artist drew inspiration from spider webs and the idea of a ‘spider‐silk future’. He created intricate, web‐like installations and sculptures using various materials, reflecting the complex geometry found in spider webs. Likewise, Raqib Shaw, a contemporary painter from India, has created stunning and intricate paintings featuring spiders as part of his elaborate and fantastical scenes. Spider silk has also been used to create art, as in Nicholas Godley and Simon Peers' intricate textile woven from the golden silk of over one million nephilid spiders (Mammola *et al*., 2017).

The framing of spiders in contemporary cultures, e.g. through artistic expression, contribute to our collective societal view of spiders, and is implicated in improving the reputation of spiders or reducing their presentation as terrifying creatures. Tapping into the societal perception of spiders may help achieve better conservation outcomes and reduce the harmful effects of arachnophobia (see Section V.5).

### (4) Educational

Experiences with spiders in environmental education not only provide a unique opportunity to enhance knowledge and promote positive attitudes towards nature and its conservation (Albo, Montes De Oca & Estavan, 2021; Hebets *et al*., 2018) but can also play a crucial role in the classroom. Studies have shown that incorporating spider activities in scholarly programs enriches students' understanding of ecosystem processes (Wagler & Wagler, 2018) and serves as a platform for discussing and debating moral issues related to using animals for educational and scientific purposes (Karlan, 2017).

In educational settings outside of formal classrooms, experiences with spiders are also impactful. A community‐based informal science program called ‘Eight‐Legged Encounters’ incorporates interactive modules that blend unique original artwork, engaging volunteers, and numerous creative games, crafts, and activities about the biology of spiders. Following the event, participants have expressed increased interest in and appreciation for spiders (Hebets *et al*., 2018). Similarly, there is an increasing number of insect fairs hosted by universities around the world (e.g. Insectapalooza at Cornell University), and exhibitions such as ‘Spiders Alive’ (in the American Museum of Natural History in New York City) featuring spiders. The interactive exhibition in the Australian Museum ‘Spiders – From Fear to Fascination’, and the recent ‘Spider Pavilion’ exhibition with live specimens in the Natural History Museum of Los Angeles County offer visitors the opportunity to interact with and see these animals in a positive light and raise public awareness. In Mexico, certified tarantula breeders also engage with students to present the life history of endemic tarantulas and the importance of preserving them.

Citizen science is an approach that has yet to be fully explored by spider enthusiasts. Initiatives such as bioblitzes (Pollock *et al*., 2015) or the SpiderSpotter app and experiential educational opportunities offered through ‘bug camps’ can help to educate and involve people in conservation. Data submitted to photo‐sharing platforms like iNaturalist increasingly contribute to studies focused on the ecology, conservation, or invasion biology of charismatic insect taxa such as butterflies, bees, and dragonflies (Skvarla & Fisher, 2023). Spiders have the potential to attract attention, particularly for species that are both large and easily recognisable through amateur images (Deitsch *et al*., 2024). Indeed, tarantulas (Theraphosidae), velvet spiders (Eresidae), black widows (Theridiidae), golden orb‐weavers (Nephilidae) and many other easy‐to‐identify species are commonly reported using citizen science platforms and other community science initiatives, which help to generate useful scientific information (e.g. Campbell & Engelbrecht, 2018; Hart, Nesbit & Goodenough, 2018; Chuang *et al*., 2023), connect everyone interested in spiders, and attract the interest of people unfamiliar with or wary of them.

Addressing societal and/or cultural biases that might favour charismatic species over ecologically significant ones like spiders poses a challenge for science and society. Misconceptions perpetuated by media sensationalism further compound this issue (Mammola *et al*., 2022), potentially influencing important decisions related to conservation efforts. Indeed, evidence suggests that attention towards species and the allocation of resources for conservation is significantly influenced by societal values and perceptions of nature (Díaz *et al*., 2015, Mammola *et al*., 2023*a*). Consequently, there is a tendency to prioritise charismatic and culturally iconic species, usually vertebrates such as mammals and birds, over equally threatened but less‐popular ones like spiders (Cardoso *et al*., 2011; Cardoso 2012; Mammola *et al*., 2020). This bias results in certain groups benefiting from sustainable management, public support for conservation actions, and successful fundraising efforts, offering enhanced protection against extinction – a phenomenon encapsulated by the ‘biocultural aspect’ of species extinctions (Ladle *et al*., 2023). Efforts in science communication and policy‐making are crucial to rectify these misconceptions and integrate spiders into conservation frameworks effectively (Milano *et al*., 2021). While applying all these tools, it is essential to evaluate rigorously the effectiveness of various science communication approaches in shaping people's attitudes and values. This can be achieved by borrowing techniques and methods from the social sciences, ensuring the use of the most effective communication strategies for the target audience.

### (5) Mental health

Specific phobias are among the most common mental disorders, making them a major health concern. The widespread mental health impact of specific phobias was demonstrated in a study (Wardenaar *et al*., 2017) covering 22 countries which found a 12‐month prevalence of specific phobias ranging from 2.6 to 12.5% (average 7.4%), with 18.7% of subjects reporting several impairments. Of all the subcategories of phobias, animal fear had the highest prevalence (3.8%). Within animal phobias, spiders often elicit greater fear and disgust responses than other arthropods (Gerdes, Uhl & Alpers, 2009). Evidence indicates that arachnophobia is the most common biophobia (Correia & Mammola, 2024). It may impair different aspects of the sufferer's life and the lives of those around them (Norberg *et al*., 2024), and can contribute to broader environmental and health issues, e.g. through the overuse of insecticides and repellents in households (Gish, Hisano & Soga, 2024) and other forms of direct persecution (e.g. Gott, 2020). Hence, it is critical to deepen understanding of the mechanisms underlying the manifestation and spread of arachnophobia as well as evidence‐based treatment options. Available evidence suggests that exposure therapy is the most effective treatment for specific phobias (Odgers *et al*., 2022), including biophobias (Norberg *et al*., 2024). Extended treatment sessions (2–3 h) combined with consistent daily exposure can result in significant fear reduction (Odgers *et al*., 2022). Conversely, there is limited evidence supporting the effectiveness of medication (Diemer *et al*., 2013). Although fear can be addressed through imagination, virtual reality, or computer‐assisted programs, real‐life exposure is generally the most effective approach in the short term (Wolitzky‐Taylor *et al*., 2008). Thus spiders themselves represent the most effective tool for treating arachnophobia, which can be efficiently provided to large groups of participants through programs at museums and zoos (Li, Newby & Graham, 2020). Given the high prevalence of arachnophobia among biophobias, we suggest there is wide scope for future experimentation in this area, with research findings potentially generalising to other phobias.

### (6) Sense of place

The presence of endemic spider species in most regions worldwide not only highlights their rich biodiversity, but also offers a unique opportunity for fostering a sense of responsibility and connection with natural heritage among local communities. This connection can be forged through collaboration and engagement with people sharing their habitats with these spiders, as suggested by Forristal, Lehto & Lee (2014). This intrinsic value is often reflected in the naming of these spiders. In fact, the choice of species names frequently reflects the places where these spiders are found, especially when they are endemic to particular regions. Mammola *et al*. (2023*b*) found that the etymology of around 27% of described spider species referenced a geographic place. This practice may cultivate a sense of pride and stewardship among local people, instilling a deeper appreciation for the unique biodiversity surrounding them. This way spiders (as other organisms) might be used as flagship species, to protect other species and their habitats.

### (7) Scientific discovery

Spiders are especially suited for answering general and pressing questions in ecology, evolution, biogeography, physiology, development, and behaviour. Two species – *Cupiennius salei* (Keyserling, 1877) and *Parasteatoda tepidariorum* (C. L. Koch, 1841) – have firmly established themselves as laboratory models to answer evolutionary, sensory, and developmental questions in arachnid science and beyond (Barth, 2002; McGregor *et al*., 2008). Beyond model species, spiders have been used as case studies for the development of new theories or methods whose results are later applied to other taxa and often become standards (Hesselberg & Gálvez, 2023). In the area of island biogeography, for example, spiders have been used to test the effect of native habitat area in explaining island richness (Cardoso *et al*., 2010) and to demonstrate the importance of directional dispersion in community composition (Carvalho *et al*., 2015). They have been used to test the applicability of novel ways to measure beta diversity (Carvalho, Cardoso & Gomes, 2012) and phylogenetic or functional diversity (Cardoso *et al*., 2014*a*,*b*). The increased availability of sequencing has enabled the design of protocols to define food webs from predator gut contents, and arachnids are excellent model predators for such ecological applications (Krehenwinkel *et al*., 2017; Uiterwaal & DeLong, 2020). The effects of drugs on behaviour were also studied in a classical experiment of web‐building behaviour under the effect of different types of substances (Witt, 1971).

The predatory capabilities of spiders, including both sensory capacities for detecting prey and physical prowess for rapid and effective attacks, creates unique challenges for potential mates during reproductive interactions and thus makes spiders excellent models for sexual selection research (Herberstein & Hebets, 2013; Herberstein *et al*., 2014). Indeed, spiders have been argued to be good models for behaviour generally, with a few focal genera appearing frequently in published behaviour studies (*Schizocosa, Nephila, Argiope*; Herberstein *et al*., 2014). *Schizocosa* research has led to significant new insights regarding the evolution and function of multimodal communication (Hebets & McGinley, 2019), while behavioural research on *Nephila* and *Argiope* has focused on sexual selection and sexual conflict (Herberstein *et al*., 2014). Other orbweaver spiders such as the European garden spider, *Araneus diadematus* Clerck, 1757, have been used as model organisms to study construction behaviour, cognition, behavioural flexibility, and animal contests (e.g. Herberstein, 2011). Spiders have also proved to be ideal models to test hotly debated hypotheses in evolutionary biology and behavioural ecology (e.g. extended phenotype concept; niche construction perspective; extended cognition hypothesis) (e.g. Wolff *et al*., 2021), and to understand the evolution of sociality (Herberstein, 2011).

### (8) Monitoring of biodiversity and ecosystem health

Spiders can serve as indicators of diversity for other taxa or environmental health. Changes in spider populations and species composition have been used to reflect and predict alterations in habitat extent, quality, pollution levels, and other environmental factors. For example, spiders have been proposed as excellent bio‐indicators for threatened ecosystems including island forests (Cardoso *et al*., 2010), coastal dunes (Ghione *et al*., 2013), inland sand ecosystems (Buchholz, 2010), bogs (Scott, Oxford & Selden, 2006), grasslands (Schwerdt, de Villalobos & Miles, 2018), caves (Doran *et al*., 1999), and Arctic tundra (Viel *et al*., 2022). Gregorič *et al*. (2022) successfully used spider webs to extract environmental DNA (eDNA) and create community profiles, with no need to resort to expensive field equipment, labour or killing the target species. A recent work by Melcher *et al*. (2024) revealed that spiders are efficient natural DNA samplers, with gut content metabarcoding revealing a similar community composition to that identified by traditional metabarcoding, generating an overview of trophic interactions and dietary ecology in arthropod communities, and allowing reconstruction of historical diets.

Spiders are also well established as indicators of chemical contaminants in aquatic ecosystems (Chumchal *et al*., 2022) and heavy metal pollution (Yang *et al*., 2016). Ines *et al*. (2024) found that a funnel‐shaped web spider [*Aglaoctenus lagotis* (Holmberg, 1876)] living alongside highways accumulated up to six times more polycyclic aromatic hydrocarbons (PAHs) compared to those along other road types. While promising, most spider‐based biomonitoring approaches remain case studies with limited applications at broad spatial or temporal scales. Identifying which spider species and technological approaches to incorporate into long‐term biomonitoring programs is still an open question, requiring further research and testing.

## VI. A RESEARCH ROADMAP

The vast diversity of described (and undescribed) spider species offers limitless opportunities for discovery and innovation. The >53,000 known species (and maybe twice as many still undescribed) allow researchers to focus on new organisms that likely will offer possibilities beyond the traditional taxa. Expanding our focal taxa will lead to new discoveries and innovations and may foster new ways of thinking about both old and new scientific problems.

Despite significant progress in spider research, critical gaps in fundamental knowledge persist, and addressing them is essential for advancing conservation efforts (Branco & Cardoso, 2020). There is limited understanding of the basic ecology of many families, including their distribution and population dynamics, particularly in the Global South where highest diversity and scarce resources often coincide. These deficiencies hinder our ability to conduct accurate extinction risk assessments, design effective conservation strategies, and establish levels of sustainable exploitation of species. Similarly, data on the global trade of spiders remain sparse due to the lack of regulation and monitoring (Cardoso *et al*., 2024).

While these gaps are significant, it is important to recognise that we already have many tools at our disposal to address them. Spiders have the most comprehensive and efficient taxonomic database of any large taxon, with their nomenclature and relevant literature being updated almost daily (World Spider Catalog, 2025). Functional species traits are recorded in the World Spider Trait Database, a constantly growing resource for ecological and comparative studies (Pekár *et al*., 2021). An R package (*arakno*) facilitates connection to and data retrieval from these databases, and further allows the cleaning, use, and adaptation of taxonomic, geographic, functional, and phylogenetic data (Cardoso & Pekár, 2022). A robust phylogenetic tree including all families and many genera from around the world is available (e.g. Kulkarni, Wood & Hormiga, 2023). An optimised and standardised sampling protocol adapted to different habitat types has been adopted worldwide, allowing analyses of spider assemblages from local to global scales (Cardoso, 2009; Malumbres‐Olarte *et al*., 2017). These resources place arachnologists in a unique position to advocate for tailored and effective spider research and conservation. There is a significant opportunity for ecosystem services provided by spiders to pave the way for new lines of research, inspire nature‐based solutions, and step into the spotlight.

### (1) Quantifying ecosystem service provision

To advance spider research and recognition, it is crucial to assess and quantify all the ecosystem services spiders provide – which can be achieved through their valuation *via* market‐based approaches. Although there is an inherent risk in discussing biodiversity in monetary terms (Temel *et al*., 2018), this approach is particularly effective for showcasing the importance of spiders to audiences beyond academia (Brander *et al*., 2024). Economic valuation of spider services is currently only available in the area of pest control. For example, the economic value of spiders was estimated to be higher than that of other natural enemies of pests of cotton (Sterling, Dean & Abo El‐Salam, 1992), with the benefit estimated to be between US$ 1–16/ha. Promotion of spiders instead of pesticides in pest control will contribute to increased crop quality leading to better marketability due to increasing demand for environmentally friendly products. In addition, it will foster biodiversity of agroecosystems and surrounding habitats.

It should be possible to quantify the monetary value of provision of other services using rough calculations of monetary savings. For example, if biodiversity data for a location can be gathered efficiently *via* eDNA from spider webs, this could potentially save surveying time. The value of the person‐hours saved could be easily quantified and reported in publications. For breakthroughs in biomimetic technologies or novel medicine, the value of these innovations can be easily calculated, either expected or effective depending on the stage of development, from pre‐ to post‐commercialisation.

Beyond the economic value, benefits to health could be quantified in the future. Comparisons could be made between target groups consuming products of conventional agriculture *versus* integrated or organic agriculture that uses bio‐insecticides derived from spider venom or spiders as biological control agents. Human wellbeing itself can be quantified, with well over 100 indicators having been proposed (Loveridge *et al*., 2020). Many of these could be tested in different settings using spiders, for example, by surveying participants before and after visiting an exhibition depicting spiders or how important pet keeping or spider‐themed culture are for multiple target groups. All these options to quantify the benefits spiders provide are still to be explored and could constitute productive avenues for future research work bridging the gap between science and application.

### (2) Potential additional services

Some potential services that might be offered by spiders are yet to be researched. Burrowing spider species, for example, might play an important role in soil formation by creating tunnels and burrows and burying prey or remains underground (e.g. Decae, 2005). These activities may enhance soil aeration, fostering crucial microbial activity and nutrient cycling essential for plant growth. Additionally, their excavation activities aid in mixing soil layers, facilitating organic matter breakdown and nutrient dispersion throughout the soil profile, enriching it with essential elements vital for vegetation. Some above‐ground spiders also exhibit morphological characteristics, such as a hairy body covering, which could theoretically enable them to collect pollen while visiting inflorescences. While observations indicate that these spiders occasionally consume nectar (Nyffeler, Olson & Symondson, 2016), direct evidence supporting their role in pollination is yet to be explored.

It is crucial to acknowledge that many of the identified services throughout this review constitute potential services, including services that might eventually be commercially exploited. These represent promising applications that may or may not lead to tangible services in the future, depending on technological advances, profitability, scalability, and more. As we navigate the frontier of spider‐inspired innovations, it is essential to approach these potential services with a balance of enthusiasm and caution. Time will reveal the extent to which these applications can be harnessed to improve human well‐being.

## VII. CONCLUSIONS

(1) Spiders are ubiquitous and abundant predators within terrestrial ecosystems that often face an unjustly negative reputation. Yet, these fascinating creatures play a crucial role by offering numerous ecosystem services vital for both the planet's future and human well‐being. In this work, we delve into the diverse range of services provided by spiders and their potential to inspire or directly contribute to nature‐based solutions.
(2) Provisioning services supplied by spiders cover a broad spectrum of applications. They encompass the versatile utilisation of silk‐like and other materials, serving as a source of inspiration for biomimetic technology, contributing to medicines derived from their venom, hemolymph or silk, acting as eco‐friendly bio‐insecticides replacing synthetic chemicals, providing food for various human communities worldwide, and becoming unconventional yet increasingly valued pets.
(3) Regulatory services by spiders play vital roles in agricultural settings by controlling pests, curbing insect‐mediated pathogen spread, and managing invasive species.
(4) Spiders offer extensive supporting services, such as nutrient cycling through organic matter breakdown, serving as vital food sources for predators, or crafting habitats for other organisms.
(5) Spiders hold substantial cultural and spiritual significance globally. They are integral to traditional medicine practices, inspire contemporary culture, offer educational value, enhance mental health, evoke a sense of place, contribute to scientific discoveries, and can be used for biodiversity and ecosystem health monitoring.
(6) While many of the services by spiders are well documented and studied, others hold untapped potential. By using nature's inherent opportunities, these natural solutions provide avenues to tackle challenges like biodiversity loss and societal needs. For this potential to be fully expressed, we need rigorously to quantify and understand the ecosystem services provided by spiders.

## AUTHOR CONTRIBUTIONS

P. C.: conceptualisation, investigation, writing – original draft, writing – review & editing; S. P., K. B., A. C., C. S. F., E. A. H., Y. H., T. H., O. M., R. M., C. S. & S. M.: investigation, writing – original draft, writing – review & editing; J. M.‐O., J. W.: investigation, visualisation, writing – original draft, writing – review & editing.
